# Evaluation of Risk Determinants and Molecular Characterisation for Non-Primate Hepacivirus Infection in Turkish Horses

**DOI:** 10.3390/pathogens14121256

**Published:** 2025-12-08

**Authors:** Mustafa Ozan Atasoy, Turhan Turan, Remziye Özbek, Hakan Işıdan, Rania F. El Naggar, Ahmed F. Afify, Mohammed A. Rohaim

**Affiliations:** 1 Department of Veterinary Virology, Faculty of Veterinary Medicine, Cumhuriyet University, 58140 Sivas, Türkiye; mozan@cumhuriyet.edu.tr (M.O.A.); tturan@cumhuriyet.edu.tr (T.T.); remziyeozbek@cumhuriyet.edu.tr (R.Ö.); hisidan@cumhuriyet.edu.tr (H.I.); 2 Department of Virology, Faculty of Veterinary Medicine, University of Sadat City, Sadat 32897, Egypt; rania.elnagar@vet.usc.edu.eg; 3 Virology Research Department, Animal Health Research Institute (AHRI), Agriculture Research Center (ARC), Giza 12618, Egypt; ahmed.afify@ahri.gov.eg; 4 Department of Virology, Faculty of Veterinary Medicine, Cairo University, Giza 12211, Egypt

**Keywords:** non-primate hepacivirus, equine hepacivirus, molecular detection, 5′ UTR, NS3, NS5B, sequence analysis

## Abstract

The genus *Hepacivirus* (HCV) has long been a dynamic group, increasing its number by myriads of species collectively referred to as non-primate hepaciviruses (NPHVs). NPHV exhibits a broad hepatotropism and is often attributed to chronic infection in horses and dogs. However, recent studies and meta-analyses on NPHV in horses have remained inconclusive regarding the determination of risk factors for infection. Therefore, our main goal was to investigate the frequencies and molecular characteristics of NPHV infection linked to geographical location, horse breeds, genders, and ages. For this purpose, we tested the positivity of 152 samples collected from ten cities in Turkey by conventional PCR, targeting the highly conserved 5′ UTR sequence, and compiled our results with pedigree data of horses for statistical analyses. We further implemented sequencing of the 5′ UTR, NS3, and NS5B regions and used in silico approaches to evaluate the characteristics of our novel isolates. Our results revealed a high incidence of NPHV infection (52.3%), which fluctuated among cities (26.1–75.0%), and comparative statistical analyses indicated that age and geographical region, together with managemental alterations, could be key determinants in NPHV infection. Furthermore, our phylogenetic analyses and 3D modelling approaches on genomic sequences revealed fundamental characteristics of novel NPHV strains. In conclusion, our study contributed to providing a better understanding of NPHV distribution and revealed some hints on diagnostics and good practices for disease prevention.

## 1. Introduction

Hepacivirus (HCV) is a positive-sense RNA virus classified in the *Hepacivirus* genus of the family *Flaviviridae* [1]. Numerous studies have demonstrated the presence of the HCV in various species, including mice, bats, cattle, primates, dogs, and horses [2,3,4,5,6]. The International Committee on Taxonomy of Viruses (ICTV) has classified hepaciviruses (HCVs) into 14 distinct species based on phylogenetic analyses (https://ictv.global/taxonomy (accessed on 1 December 2025)). Among these, equine hepaciviruses and canine hepaciviruses have been grouped into a single species, *Hepacivirus equi* (EqHV), which shares a close genetic relationship with human HCVs [7]. As such, they have been included in the hepacivirus A species and often referred to as non-primate hepaciviruses (NPHVs), indicative of an adaptation process likely arising from cross-species transmission [8].

NPHV-related diseases affect horse populations worldwide and are mainly transmitted through vertical, parenteral or iatrogenic routes [9,10,11,12]. Unlike HCV in humans, which often leads to chronic infection, NPHV in horses more commonly results in acute infection [13]. NPHV infection has been detected in horses with severe hepatitis syndrome (often referred to as Theiler disease); however, clinical onsets of acute and experimental infections are limited to mild hepatitis [14,15,16]. Several reports from NPHV-infected horses have found a slight increase in liver function test values, indicating subclinical infection [15,17,18]. NPHV has been detected in clinically healthy horses; therefore, the potential contribution of virus to liver disease is often overlooked [7,12]. In addition, various risk factors, such as the age, sex, or breed of the horse, have been determined as predisposing factors for NPHV infection [19].

The genome of NPHV comprises a single open reading frame (ORF) encoding sequences for structural (core, E1, and E2) and non-structural (p7, NS2, NS3, NS4A, NS4B, NS5A, and NS5B) proteins, along with two untranslated regions (UTRs) in the 5′ and 3′ ends. Several studies have identified various subtypes through phylogenetic analyses of partial sequences in the 5′ UTR, NS3, or NS5B regions, underscoring NPHV’s exceptionally well-conserved genome compared to other hepaciviruses [17,20,21]. The 5′ UTR sequence is considered an essential element for genotyping and molecular evolutionary studies of non-primate hepaciviruses [22]. Furthermore, two non-structural proteins, namely, NS3 and NS5B are minimally required for the successful replication of viral RNA [23]. The NS3 gene encodes a multifunctional non-structural protein that primarily serves as a serine protease, helicase, and nucleoside triphosphatase. This protein plays a crucial role in viral processes, including the post-translational processing of viral polyproteins, and facilitates the virus’s evasion of the host’s immune system by deactivating key innate immune system components, such as MAVS and TRIF [24]. Furthermore, studies have suggested that NS3 protein plays a key role in host switching [25,26].

Another important non-structural gene, NS5B, encodes RNA-dependent RNA polymerase (RdRP), which is essential for catalysing viral RNA replication. The NS5B protein is widely regarded as a significant target for antiviral drug development [27,28]. Furthermore, extensive phylogenetic studies indicate that the NS5B gene undergoes random recombination events, contributing to the emergence of novel HCV variants [29,30]. N5B is also responsible for modulating critical functions for the virus, including the processing post-translational modifications of viral polyproteins, or assists the virus in evading the host’s immune response by deactivating innate immune system components, such as MAVS and TRIF [24].

According to TURKSTAT, Turkey’s registered horse population is currently estimated at approximately 80,000, showing a declining trend over the past two decades [31]. In this context, raising a healthy generation of horses is paramount, and continuous monitoring of infectious diseases plays a critical role in sustaining the equine industry. Non-primate hepaciviruses (NPHVs) have been largely overlooked to date; therefore, we aimed to investigate the presence of this virus in horse herds in Turkey, with reference to individual risk factors and regional differences, and to provide fundamental molecular insights based on deep molecular and bioinformatic analyses for further characterisation of the viruses.

## 2. Materials and Methods

### 2.1. Acquisition and Processing of Field Samples

The study primarily selected ten provinces (Canakkale, Istanbul, Ankara, Nevsehir, Kayseri, Sivas, Tokat, Hatay, Erzurum and Mus) spanning four geographical regions, with all serum specimens gathered from private stud farms or individual horse breeders during 2021. 152 samples were randomly collected within the regions following approval from the Sivas Cumhuriyet University Animal Experimentation Local Ethics Committee (Decision No: 65202830-050.04.04-560 Date: 1 June 2021). For each equine, age, breed, and sex were documented for subsequent statistical evaluation. All horses were clinically healthy, showing no apparent signs of infection. For serum sampling, 5–10 mL venous blood samples from the jugular vein of horses using an 18-gauge needle and transferred into plain tubes by the assistance of local veterinary practitioners. The samples were transported to the laboratory within 24 h under cold chain conditions and aliquoted into 1.5 mL microfuge tubes. After that, the tubes were centrifuged at 2000× *g* for 5 min to separate the sera, which were then stored at −80 °C until RNA-isolation will take place.

### 2.2. RNA Isolation, Reverse Transcription and PCR Assay

Total RNA was extracted from 200 μL serum aliquots using the Total RNA extraction kit (Vivantis, Subang Jaya, Malaysia) following the manufacturer’s instructions. A total of 25 μL of RNA samples were eluted and quantified using a nanometre (Denovix, Wilmington, DE, USA). Subsequently, total RNA extracts from each sample were reverse transcribed by using RevertAid Reverse Transcriptase Kit (Thermo, Waltham, MA, USA) to yield cDNA. The nested primer sets used in study were previously described by Lyons et al. (2012) [17]. For the initial screening of the serum samples, a nested PCR assay was employed based on the highly conserved 5′ untranslated region (UTR) of the viral genome, with modifications in the protocol. In the first round of the PCR, 12.5 μL of 2X PCR Master Mix (Thermo, Waltham, MA, USA), 200 nM of EQ5-UTROS and EQ5-UTROAS primers, and 250 ng of cDNA were mixed and completed to 25 μL of reactions. The thermocycler conditions were adjusted as follows: initial denaturation at 95 °C for 3 min, followed by cycling (35 cycles at 95 °C for 20 s, 50 °C for 30 s, and 72 °C for 1 min, with a final extension step at 72 °C for 5 min. Next, 5 μL from each reaction was used to perform a second round of PCR assay, employing a second pair of primer sets, EQ5-UTRIS and EQ5-UTRIAS, under the same conditions. Positive samples were determined by running the second round of PCR reactions in the agar gel electrophoresis, where the presence of a 231 bp band under UV light indicated positivity.

Positive samples underwent further nested PCR amplification using primer sets targeting two non-structural genes, NS3 and NS5B. The PCR master mixes were prepared in the same manner as for the 5′ UTR. The cycling conditions for both rounds comprised an initial denaturation step of 95 °C for 3 min, followed by 35 cycles at 95 °C for 20 s, 50 °C for 30 s, 72 °C for 1 min; and a final extension step at 72 °C for 5 min. The second round of nested PCR reactions for NS3 and NS5B were subjected to agarose gel electrophoresis, screened under the transilluminator, and intense bands corresponding to the respective genes were confirmed as positive.

### 2.3. Sample Sequencing

Our primary objective was to characterise the molecular features of NPHV detected in warm-blooded horses from the Central Anatolian region, together with additional cases from the southeastern province of Hatay. Therefore, positive samples were randomly selected from Middle Anatolian and Hatay cases for sequencing. For this purpose, positive bands were excised from the 1% agarose gel under the transilluminator and recovered using a gel purification kit (AmbiClean Kit, Vivantis, Subang Jaya, Malaysia), according to the manufacturer’s instructions. Bidirectional sequencing was conducted twice utilising the BigDye Terminator Cycle Sequencing Kit (Applied Biosystems, Foster City, CA, USA) with an automated sequencer (ABI 3100; Applied Biosystems, Foster City, CA, USA). Raw data, featuring robust coverage (>90%), from both forward and reverse reads were assembled for each sample. Further details of the sequenced samples are provided in Supplementary Material (Supplementary Table S1).

### 2.4. Multiple Sequence and In Silico Analyses

Raw chromatograms from bidirectional Sanger sequencing were assessed using Phred scores along with sequencing statistics (confidence mean, expected errors, and error-free odds). Low-quality terminal regions (Phred < Q20) were trimmed, and bases with Phred ≥ Q20 were retained for consensus building. Forward and reverse reads were assembled, with manual inspection of chromatograms to resolve ambiguous or low-quality positions. Then, reads belonging to each strain were first assembled and then aligned with the reference strain NZP1 (KP325401) to locate positions and deduce amino acid sequences using Geneious Prime software (version 2026.0.1) [32]. A total of nine sequences from the 5′ UTR (198 to 203 base), five sequences from NS3 (175 base/57 aa), and four sequences from NS5B (308 base/102 aa) were successfully retrieved. To predict the secondary structures of 5′ UTRs, minimum free energy (MFE) structures were generated using the RNAfold server [33]. Phylogenetic trees were constructed using PhyML (v.3.3.20180621) [34]. The primary criteria for selecting previously reported strains for phylogenetic tree construction were the genetic diversity of the detected strains, initially assessed using the BLASTn tool (v.2.16.0; https://blast.ncbi.nlm.nih.gov/), along with their geographical proximity to properly evaluate potential associations.

The Bayesian Information Criterion (BIC) was used to determine the best-fit evolutionary model, which was then applied to construct the most probable phylogenetic trees [35]. Recombination analysis was performed using RDP5 software (version Beta 5.77) [36] on each nucleotide sequence dataset, applying the default settings (*p*-value cutoff = 0.05) across the algorithms RDP, GENECONV, Chimaera, MaxChi, BootScan, SiScan, and 3Seq.

Hypothetical 3D models of NS3 and NS5B were constructed using the SWISS-MODEL Repository [32], based on the NZP1 reference sequence (NC_038425.1) as a target sequence. The best-fitting models were selected and evaluated using GMQE and QMEANDisCo scores to assess overall reliability and accuracy, MolProbity scores to identify steric conflicts, and Ramachandran analysis to examine stereochemical quality [37], all of which were briefly given in Supplementary Material (Supplementary Table S2). Deduced amino acid sequences from each dataset were aligned using MAFFT to determine sequence homology and identify conserved regions, which were then mapped onto each model.

### 2.5. Statistical Analyses

PCR results of the data were combined with each horses’ individual records which consist of breeds (Indigenous, Arabian, Thoroughbred, Pony and Haflinger), gender, collection area and ages. The relationship between age and infection status was analysed in R Studio (v.2024.09.0+375) using logistic regression, with age as the predictor, and a non-parametric test, Odds ratios with 95% confidence intervals were calculated to measure how age affected the likelihood of testing positive. Age data were further classified into three groups based on the data distribution, 0–6 years (*n* = 61), 7–9 years (*n* = 45), and ≥10 years (*n* = 46), and the logistic regression analysis was repeated. Additionally, a Mann–Whitney U test was used to compare the age distributions between positive and negative groups without assuming normality.

To assess the association between gender and infection status, chi-square tests of independence were conducted, with odds ratios and 95% confidence intervals reported where appropriate. One-way ANOVA was conducted to examine age differences between participants from 6 cities (*n* = 150), after excluding Mus and Tokat. Games–Howell post hoc tests were used due to unequal variances. To assess the independent associations between potential risk factors and infection, multivariate logistic regression analysis was performed. Model fit was evaluated using the Hosmer–Lemeshow goodness-of-fit test, and the proportion of explained variance was assessed using Nagelkerke R^2^. Statistical significance was set at *p* < 0.05.

## 3. Results

### 3.1. Occurrence of NPHV in Serum Samples

**Figure 1:**
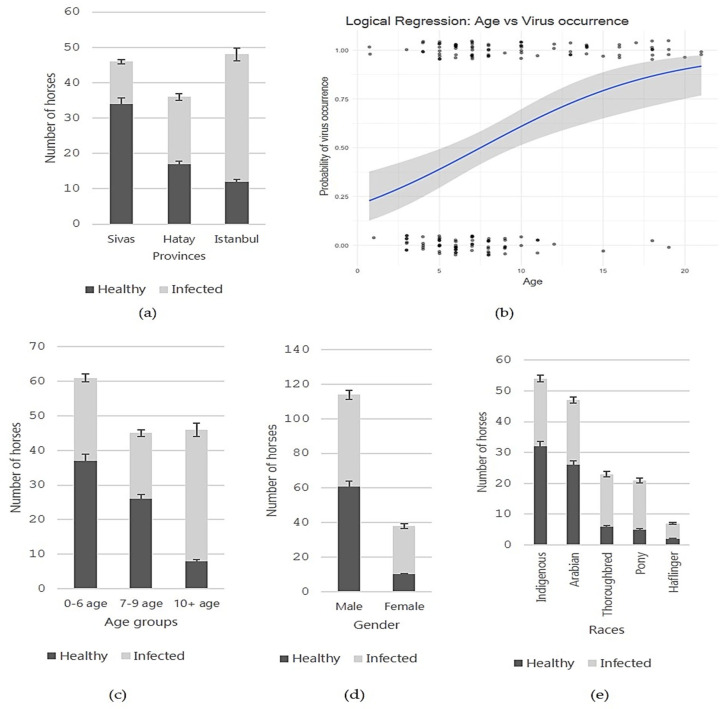
Statistical analysis of NPHV infection in horses. (**a**) Distribution of NPHV-positive and -negative animals across the three provinces with the greatest sample representation. (**b**) Logistic regression demonstrating a positive association between age and NPHV infection likelihood; the blue line indicates the increasing trend with age. (**c**–**e**) Bar charts illustrating how NPHV infection is distributed across different age groups (**c**), genders (**d**), and breeds (**e**). Results showed marked variation by geography, age, sex, and breed: positivity was greatest in Istanbul (75.0%), in horses aged 10+ years (82.6%), in females (73.7%), and among Pony and Thoroughbred breeds (76.2% and 73.9%). Logistic regression further demonstrated a significant positive association between age and infection likelihood.

Amongst 152 serum samples obtained from horses across 10 distinct provinces, 81 samples (52.3%) were found to be positive for NPHV based on nested PCR results targeting 5′ UTR which was the most conserved region. The prevalence in Sivas was relatively lower at 26.1% (12/46), while in Hatay, it was determined to be 52.7% (19/36), and in Istanbul, it notably surged to 75.0% (36/48) (Figure 1a). Additionally, four out of seven samples from Balikesir, three out of five samples from Kayseri, all three samples from Ankara and Erzurum, and one sample from Mus tested positive for NPHV.

The data were further analysed to determine whether age was linked to infection status (positive/negative) using both logistic regression and a non-parametric test. Both statistical approaches revealed that increasing age was significantly associated with a higher probability of infection (*p* < 0.0001), as shown in Figure 1b. The results were then interpreted in relation to the animals’ ages and the regional incidence of infection. The median ages were 7.5 ± 3.4 years in Sivas, 7.0 ± 4.9 years in Hatay, and 10.9 ± 5.4 years in Istanbul. A one-way ANOVA revealed a significant difference in age between cities, F (7142) = 3.25, *p* = 0.003. Post hoc comparisons using Games–Howell showed that participants in Istanbul were significantly older than those in Sivas (*p* = 0.009) and Hatay (*p* = 0.003), but there was no significant difference between Sivas and Hatay (*p* = 1.000). Lastly, equines were categorised into three age groups, 0 to 6 years (n = 61), 7 to 9 years (n = 45), and 10 years or older (n = 46), with the prevalence interpreted as increasing across these groups, at 39.3%, 42.2%, and 53.3%, respectively (Figure 1c).

We demonstrated that gender had a significant influence on infection status. Statistical analysis revealed a strong association between gender and infection status (*p* < 0.05). The prevalence of NPHV was markedly higher in mares (28/38; 73.7%) compared with stallions (53/114; 46.5%; Figure 1d). The odds of infection were approximately 3.2 times higher in mares than in stallions (OR = 3.22, 95% CI: 1.43–7.25). Notably, A Kruskal–Wallis test showed a significant difference in age between males and females, *p* < 0.05. The median ages for mares and stallions were 7.8 ± 0.3 (95% CI: 7.1–8.5) and 10.9 ± 1.0 years (95% CI: 8.9–12.9), respectively. Indigenous (94.4%) and Arabian horses (80.9%) were predominantly male, whereas the other groups were more balanced, with only one in four horses overall being female.

On the other hand, evaluation by breed indicated that Indigenous horses exhibited a positivity rate of 40.7% (22/54), followed by Arabian horses at 44.7% (21/47), Thoroughbred horses at 73.9% (17/23), ponies at 76.2% (16/21), and Haflingers at 71.4% (5/7), as is shown in Figure 1e. Age distribution by breed was further evaluated, showing that Haflingers (13.5 ± 3.4 years) and ponies (11.3 ± 6.1 years) were generally older than 10 years, whereas Thoroughbreds (7.6 ± 4.3 years), Arabians (8.0 ± 5.1 years), and Indigenous horses (7.6 ± 2.8 years) were younger. The Kruskal–Wallis test demonstrated a statistically significant difference in age distributions among the five breeds, H (4) = 16.36, *p* = 0.003. Post hoc pairwise comparisons with Bonferroni correction indicated that the ages of Indigenous horses, Arabians, Thoroughbreds, and ponies did not differ significantly, whereas Haflingers were significantly older (*p* < 0.05). Finally, considering that the relationships between age, gender, and breed may be confounded, we performed a multivariate logistic regression including age, gender, province, and breed. The analysis showed that age (OR = 1.19, 95% CI 1.08–1.32, *p* = 0.001) and province (OR = 1.16, 95% CI 1.02–1.32, *p* = 0.025) were independently associated with infection, whereas gender and breed were not significant after adjustment. The model demonstrated a Nagelkerke R^2^ of 0.259 and good fit (Hosmer–Lemeshow test, *p* = 0.147).

### 3.2. Sequencing and Molecular Analyses

#### 3.2.1. 5′ UTR

**Figure 2:**
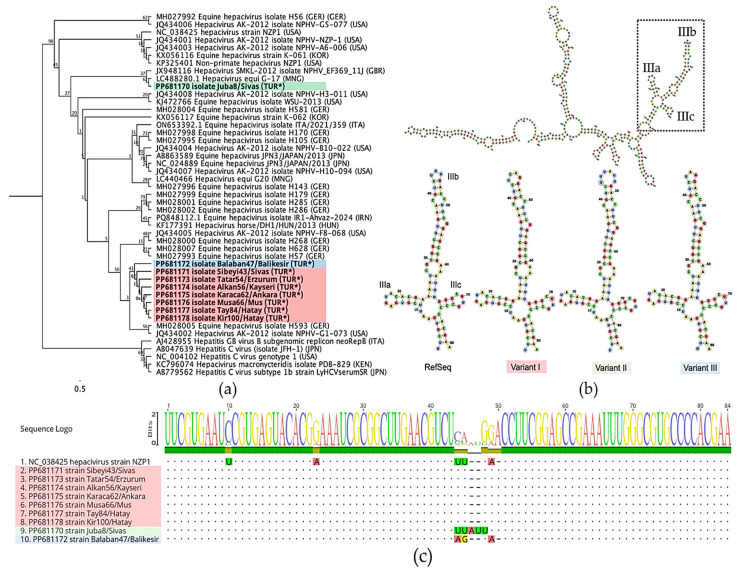
Molecular analysis based on partial 5′ UTR sequences of NPHV strains. (**a**) PhyML tree constructed using the K80 substitution model, showing that most Turkish strains, indicated with (*), cluster within a single branch, while Juba8/Sivas forms an outgroup. The tree was bootstrapped by 100 times. (**b**) Predicted RNA secondary-structure variations within the stem loop IIIb region, modelled using RNAfold [33]. (**c**) Multiple sequence alignment illustrating mutations in the IIIa–IIIc regions. Colour codes in panels (**a**,**c**) were assigned based on the structural variation analysis shown in panel (**b**).

Nine 5′ UTR amplicons, ranging in length from 198 to 201 bases, were compared with other available sequences in the NCBI databases. Multiple sequence alignment and phylogenetic analysis showed that seven of them were identical to each other and grouped into a single cluster in the phylogenetic tree (see Figure 2a). They showed a close relationship with the Balaban47/Balikesir strain (PP681172), exhibiting 99.5% nucleotide identity. In contrast, the Juba8/Sivas strain (PP681170) was identical to the G-17 isolate (LC488280.1) and diverged from the other Turkish strains, sharing only 95.57% sequence identity.

Newly identified sequences were annotated based on the reference strain NZP1 (NC_038425). The analysis indicated that the main mutations were specifically accumulated in the IIIb region, where the sequences exhibited notable variations. RNAfold analysis further confirmed that these mutations were located within the hairpin loop of the IIIb region. Turkish strains were classified into three variants (I–III) based on the structure of these hairpin loops (see Figure 2b,c).

#### 3.2.2. Partial NS3 Sequence

**Figure 3:**
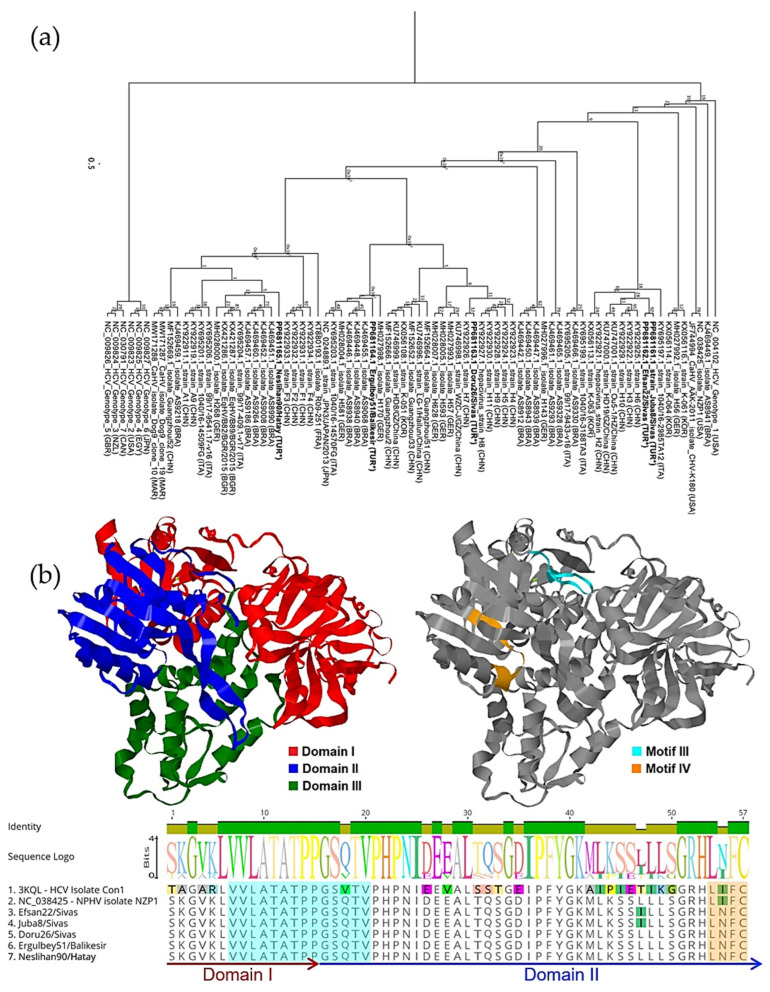
(**a**) PhyML tree based on a 172-base fragment of the NPHV *NS3* gene, constructed using the JC69 substitution model and 100 bootstrap replicates, illustrating heterogeneous distribution and clustering of Turkish strains, indicated with (*). (**b**) Hypothetical 3D structural model of reference NS3 sequences (NC_038425) highlighting defined functional domains (**left**) and the positions of sequence motifs identified in this study (**right**). Domains and motifs mapped on the 3D models are also shown in the multiple sequence alignment of 57 deduced amino acid sequences (**bottom**), showing variations among reference sequences.

Five 172 bp NS3 amplicons were aligned and compared with reference sequences from the NCBI database. Multiple sequence alignment showed that two strains from Sivas, Juba8/Sivas and Efsan22/Sivas were identical. In contrast, Doru26/Sivas differed from them, sharing only 87.28% nucleotide identity. Furthermore, Neslihan90/Hatay and Ergulbey51/Balikesir shared 86.71% identity with two Sivas strains and 89.60% identity with each other. Furthermore, phylogenetic analysis revealed that Juba8/Sivas and Efsan22/Sivas shared 96.53% identity with Chinese-origin commercial serum strains H3 (KY922922), H6 (KY922925), H10 (KY922929) and HD19/GZ/China (KU747000), forming a single branch. Doru26/Sivas was more divergent, showing 95.36% identity with Chinese-origin strains H7 (KY922926), WZC-J/GZ/China (KU746998) and H628 (MH028007). Ergulbey51/Balikesir clustered closely with Doru26/Sivas and shared 92.49% identity with a donkey-derived strain (KT880193.1). Neslihan90/Hatay had the closest identity with strain 99/17-9541.17-v16 isolated from a horse in Italy (KY695206) (see Figure 3a).

The 57 deduced amino acid sequences were compared with those of other strains by constructing hypothetical 3D models using the amino acid sequence of NS3 (YP_009664198.1) of isolate NZP1. The models were generated based on the only helicase domain (NS3h) of the HCV isolate Con1 (PDB ID: 3KQL) through SwissProt. Three domains of NS3h were determined, along with several functional motifs distributed throughout the sequence (Figure 3b). Structural analysis indicated that the 57–amino acid fragment encompasses regions corresponding to both domains 1 and 2 and includes motif III (VLATATPPGSQTV) as well as a partial sequence of motif IV (LIFCHSKKK). Motif III was highly conserved despite the presence of non-synonymous substitutions, whereas an Ile366Asn substitution was detected within motif IV, a variation less frequently observed among some reported strains including those isolated from dogs (isolate Dog9, MW171287; isolate Dog10, MW171288).

#### 3.2.3. Partial NS5B Sequence

**Figure 4:**
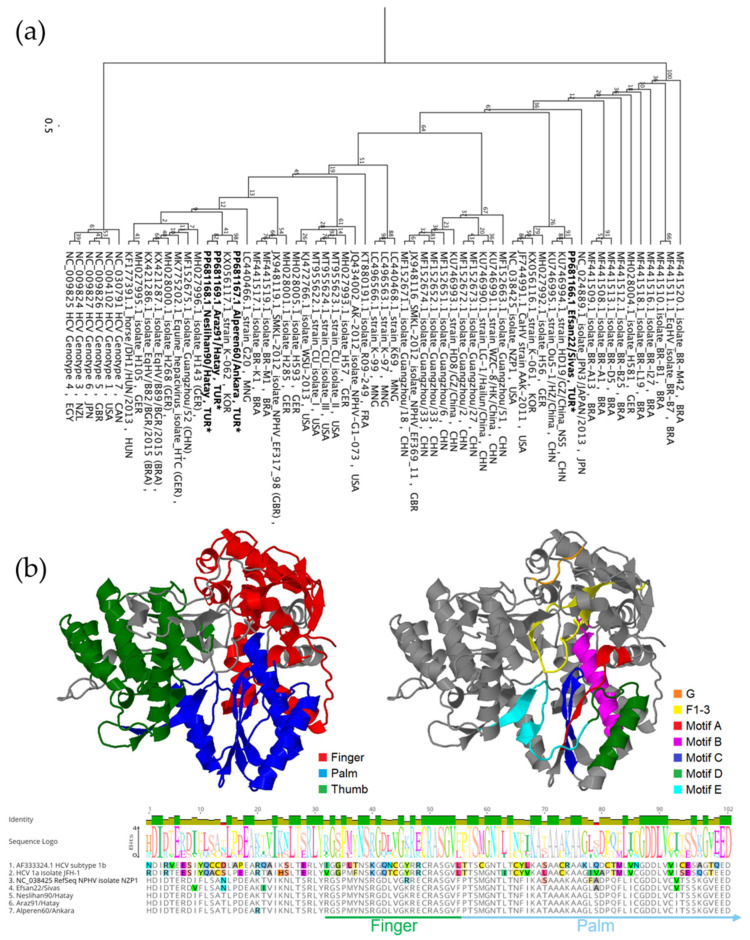
(**a**) PhyML tree based on a 308-base fragment of the NPHV *NS5B* gene, constructed using the K80 substitution model and 100 bootstrap replicates, illustrating clustering of Turkish strains, indicated with (*). (**b**) Hypothetical 3D structural model of reference NS3 sequences (NC_038425) highlighting defined functional domains (**left**) and the positions of sequence motifs identified in this study (**right**). Domains and motifs mapped on the 3D models are also shown in the multiple sequence alignment of 102 deduced amino acid sequences (**bottom**), showing variations among reference sequences.

Four 308 bp NS5B amplicons were aligned and compared with other available sequences in the NCBI database. Multiple sequence alignment revealed that the Turkish strains exhibited varying levels of identity, ranging from 83.12% to 97.73%. The Neslihan90/Hatay and Araz91/Hatay strains were closely related, as expected, showing 97.73% identity. In contrast, Alperen60/Ankara showed 92.21% identity, while Efsan22/Sivas was more distantly related, with a lower identity ranging from 83.12% to 83.77%. Phylogenetic analysis further showed that Efsan22/Sivas had the highest identity with a Chinese strain isolated from domestic horse, HD19/GZ/China (KU746994.1). Phylogenetic analysis further revealed that strains from Hatay and Ankara clustered together on a single branch, accompanied by the South Korean strain K-062 (KX056117.1) with 95.13% sequence identity, while Efsan22/Sivas formed a separate cluster primarily composed of Chinese strains (Figure 4a).

The deduced amino acid sequences were compared with those of other strains by constructing a hypothetical 3D model using the amino acid sequence of NS5B (YP_009664202.1). isolate NZP1. The models were generated based on both HCV isolate JFH-1 (PDB ID: 4WTG) and isolate BK (PDB ID: 3HHK). We successfully retrieved a partial sequence of the palm subdomain of the protein, including Motif B and Motif C. Our analyses revealed that non-synonymous mutations exist in these subdomains, with some occurring within the motifs. We detected the S298T mutation in Motif B in all Turkish strains, and the V321I mutation in Motif C of the Alperen60/Ankara, Neslihan90/Hatay, and Araz91/Hatay strains (Figure 4b).

## 4. Discussion

This study primarily aimed to determine the prevalence of NPHV in horse serum and its association with individual factors, including age, gender, breed, and geographic location. A recent comparative meta-analysis by Bezerra et al. (2022) has revealed that NPHV prevalence was 7.88% (varying between 5.23 and 11.69) worldwide [38]. This proportion was relatively found to be on the margin in Asia (16.13%; 7.79–30.43). We found a high frequency of NPHV presence, with 52.29% (81/152) of samples testing positive. Such a high prevalence is noteworthy but not unprecedented; for instance, studies have shown that Mongolia had positivity rates ranging between 39.4% and 45.0%, depending on the year, targeting on 5′ UTR site RT-PCR [39]. Similarly, in Japan, 35.48% of samples tested positive (11 out of 31) based on NS3 gene, despite the limited sample size [6]. Based on our sample size, the regional analysis was further weighted toward three provinces, Hatay, Sivas, and Istanbul, from which over 30 samples were collected. We observed a significant variation between these three provinces, ranging from 26.08% to 75.00%, clearly highlighting the regional differences in disease frequency across Turkey. Previous studies have also identified regional fluctuations in prevalence in countries such as Mongolia, Morocco, and Italy [39,40,41]. Notwithstanding, we conjectured that Istanbul, with its relatively active horse trade, is subject to more frequent international transportation and more intensive veterinary services than Hatay and Sivas. In addition, our findings revealed regional differences that may reflect variations in animal management practices and emphasised the importance of considering regional factors when evaluating NPHV frequency.

Ageing is a well-known factor influencing some viral diseases [42]; therefore, we proceeded to analyse our data by age and infection status and to assess their patterns across different regions. Statistical analysis indicated a significant association (*p* < 0.0001) between age and infection status, showing an increasing trend of NPHV occurrence with age. The relationship between age and virus incidence parameters remains inconclusive, as different studies report conflicting results. For example, Pronost et al. (2017) categorised horses into four age groups (1–4, 5–7, 8–11, and 12+ years) and observed that virus occurrence did not vary substantially across ages [21]. Similarly, a comprehensive investigation of 733 serum samples using seropositivity and molecular identification methods found no overall correlation between age and NPHV status, except in horses with a transportation history, where the probability of infection decreased by 20% with each additional year of age, indicating that younger horses were more predisposed to infection [43]. Furthermore, numerous studies across different age groups of horses have consistently demonstrated that younger horses exhibit higher infection rates [40,44,45]; however, more recent studies have reported contradictory findings [46,47,48]. Therefore, we applied the same methodology by classifying our age data into three balanced groups of roughly equal sample size (0 to 6, 7 to 9, and 10 years and above), and an increase in virus prevalence with age was identified, at 39.3%, 42.2%, and 53.3%, respectively. The prevalence of the virus in equines older than 10 years exhibited substantial variation, ranging from 13.9% in Morocco and 23.8% in Brazil to 94.0% in China [40,46,48], not least a pattern borne out by our findings to some extent. Lastly, we sought to assess the statistical significance of age variations within three cities (Istanbul, Sivas, and Hatay) and found that horses from Istanbul were older, whereas those from Sivas and Hatay had comparable ages. Thus, it is plausible that NPHV incidence increases with age, suggesting that age, together with management practices and geographical location, constitutes an important determinant.

Given the uneven distribution of horse breeds and genders across cities, we initially disregarded city-specific effects in analysing their association with NPHV occurrence due to regional breed preferences and local management practices in each city. Our study revealed a relatively high incidence of NPHV in ponies (76.2%), Haflingers (71.4%), and Thoroughbreds (73.9%), whereas indigenous breeds (40.7%) and Arabians (44.7%) showed lower incidence compared with the overall study population. Haflingers were significantly older than the other breeds, among which age variation was negligible from a statistical perspective. An initial study by Pfaender et al. (2015) investigated NPHV occurrence in 433 horses using serological and molecular methods, reporting the highest prevalence (68%) in Thoroughbreds and suggesting a possible genetic predisposition or iatrogenic transmission [13]. There are further studies giving evidence of relatively higher incidence in Thoroughbred horses [21,43,44]. A recent extensive meta-analysis by Pacchiarotti et al. (2022) inferred that Thoroughbred horses were more susceptible to NPHV infection, likely owing to their economic value and popularity, which result in intensive management, frequent veterinary care, and transportation, thereby increasing their exposure risk [19]. When considering the city factor in this study, most Ponies, Thoroughbreds, and Haflingers were from Istanbul and Hatay, while indigenous horses came from elsewhere. Altogether, Thoroughbred horses exhibited higher infection rates than indigenous horses, consistent with previous studies, while Ponies in Istanbul and Hatay showed the highest incidence of NPHV infection. Haflingers also showed a relatively high incidence, possibly due to their greater mean age.

Gender is considered a factor, with various studies showing female horses are more associated with NPHV occurrence than males. Abbadi et al. (2021) found a higher prevalence of NPHV in females in both equines and canines, pointing out that gender is an important risk factor across species [40]. Nonetheless, evidence regarding the effect of gender on virus presence remains inconclusive, with some studies supports [48,49], while others report contradictory findings [13,50]. In this study, we demonstrated a significantly higher occurrence of NPHV in females than in males, and we also detected a statistical difference in age between genders, which may have a substantial effect on our assessment. In addition, the multivariate regression analysis pointed out that gender and breed did not contribute significantly to infection risk. Interestingly, a recent comprehensive analysis of HCV prevalence across age and sex groups has recently observed a higher susceptibility among females, a finding attributable to sex-specific differences in immunological mechanisms and inflammatory responses [51]. Extending such an approach to equine populations by characterising their immunological background may enhance our knowledge of NPHV pathophysiology.

The 5′ UTR is a short sequence that plays a key role in recruiting viral RNA to ribosomes and facilitating their binding, thereby initiating translation of viral proteins [52]. Owing to its critical regulatory functions, the 5′ UTR is highly conserved within each genus of the Flaviviridae family and is commonly used as a target for molecular detection and quantification [53,54,55]. Therefore, we initially screened our samples for NPHV using conventional PCR primers targeting the 5′ UTR and then sequenced selected amplicons for phylogenetic and molecular characterisation. Multiple sequence alignments and phylogenetic analyses showed that the partially recovered 5′ UTRs of Turkish isolates were largely conserved and clustered within a central Turkish branch. In contrast, the Juba8/Sivas isolate contained several point mutations that distinguished it from this group and placed it in a separate branch. Secondary structure predictions further indicated that the main variation in 5′ UTR RNAs was localised to the circular loop of the IIIb region. Structurally, 5′ UTR contains three Stem–Loops (SLs I–III), with SLIII being necessary and sufficient for complete IRES activity [56]. In HCV, SLIIIb initiates the eIF2–eIF3–40S recruitment cascade by binding its apical loop to eIF3, and mutations in this loop can alter its interaction with eIF3 [57]. In our analysis, we detected three different variants in the apical loop of SLIIIb, which may similarly confer altered reactivity with eIF3 in each strain. Previous studies have reported divergence in the IIIb loop [7,39], and mutations in the SLIII region of the 5′ UTR should be considered in virus classification.

In vitro studies have demonstrated that NS3 helicase (NS3h) can unwind both DNA and RNA substrates by hydrolysing ATP and contributes to the assembly of hepacivirus virions [58,59,60,61]. A recent study by Ralfs et al. (2025) further demonstrated NS3h likely induces structural rearrangements on cis-acting RNA elements and unwinds terminal stem loops, thereby facilitating appropriate NS5B activity required for negative-strand genome synthesis [62]. NS3h exhibits a Y-shaped architecture consisting of two RecA-like domains (domains 1 and 2) which forms a nucleic acid–binding cleft, together with a third domain (domain 3) that facilitates nucleic acid translocation and supports enzymatic activity [63,64]. In this study, two Sivas strains (Juba8/Sivas and Efsan22/Sivas) were identical, whereas a third strain from the same region (Doru26/Sivas) showed 87.28% nucleotide identity, reflecting NS3h genetic variation among viruses in the same region. Phylogenetic analysis revealed an uneven distribution of Turkish strains, with the majority clustering alongside diverse Chinese strains derived from commercial serum samples. Notably, Ergulbey51/Balikesir, isolated from Balikesir—a region with a relatively high donkey population density—showed the closest identity to a strain previously isolated from a donkey. The 57 deduced amino acid sequences further enabled the assessment of partial sequences of domains 1 and 2 along with Motif III and partial sequence of motif IV. The fundamental functions of these motifs have been extensively investigated through mutagenesis studies in human hepaciviruses. For instance, Thr322Ala and Thr324Ala mutations were shown to result in reduced ATPase activity and decreased nucleic acid binding capacity [65,66]. We observed Motif III was well conserved within Turkish strains despite high rate of non-synonymous mutations. Motif IV (LNFC-) was only partially retrieved; nevertheless, the Ile366Asn mutation was observed in all Turkish samples, as well as in a few equines and some canine serum samples. Although motif IV has been extensively studied in human hepaciviruses [67,68,69], the functional consequences of this mutation remained unknown. Altogether, we hypothesised that motif IV might be an important determinant of NPHV biology, but this remained to be demonstrated and would require more extensive systematic mutational analyses for confirmation. Such systematic mutational analyses will not only elucidate its functional role but also reveal mechanisms underlying interspecies transmission and identify rational therapeutic strategies, given that NS3h is frequently targeted in human hepacivirus treatment [70].

Initial studies on HCV identified NS5B as the catalytic subunit of the membrane-associated replicase complex that synthesises replication intermediates of the viral genome required for transcription and translation [71,72]. Structural analyses further demonstrated that the NS5B protein adopts a canonical right-hand-like architecture, showing an analogy to a palm, thumb, and fingers, and revealed conserved motifs and essential elements required for its catalytic function [71,73,74,75]. Our study revealed genetic divergence in the NS5B gene among four viral strains, which clustered with other related strains into two distinct branches in the phylogenetic tree. These novel strains shared 83.1% to 97.7% sequence identity. The structural architecture of the NPHV NS5B protein was previously described by Albuquerque et al. (2020) [20]. Building on their findings, further analysis of 102 deduced amino acid sequences representing partial regions of the palm and finger domains, including motif B (21 amino acids) and motif C (14 amino acids), provided additional insights into their genetic variability. Motif B locates in finger domain and participates in binding the template RNA, contributes to substrate discrimination, and indirectly affects ribonucleotide triphosphate recognition by influencing the geometry of the active site [76,77]. Moreover, the motif B loop represents a key site for antiviral drug development because of its essential role in initiating RNA polymerisation [76]. Motif C, on the other hand, is composed of a β-strand–loop–β-strand structure and contains the conserved “GDD” sequence, which forms part of the catalytic site where metal ion binding occurs [78,79,80]. In this study, despite frequent non-synonymous substitutions, the amino acid sequences of motif B and motif C remained highly conserved across the analysed strains. Only the Ser298Thr substitution was observed in motif B, while Val327Ile was detected in motif C, excluding the Efsan22/Sivas strain; both substitutions have been reported in other NPHV isolates. As the functional roles of these mutations have not been investigated, we hypothesise that the overall conservation of these motifs may facilitate the design of potential chemical compounds targeting NS5B. Computational approaches for drug development targeting NS5B polymerase have been applied in HCV [81,82], and applying similar strategies by determining potential mutations in catalytically active sites together with basic in silico analyses could aid the rational design of antiviral compounds for NPHV in both equine and canine hosts.

This study has certain limitations. Although samples were collected from ten provinces representing various geographical regions of Turkey, their distribution was uneven and largely concentrated in the Marmara and Central Anatolia regions. Consequently, the epidemiological status in some areas, particularly the eastern provinces, remains insufficiently characterised. In addition, sequencing and molecular analyses primarily focused on indigenous breeds from Central Anatolia, leaving the genomic features of strains collected in Istanbul unresolved. Furthermore, our sequence analyses were based on partial genomes of two non-structural genes and the untranslated region of NPHV, while 3D analyses were conducted using whole-genome sequences of well-characterised reference strains. Potential mutations in the unsequenced regions of strains may influence viral structure and function, underscoring the need for comprehensive genomic investigation. Lastly, NS3 and NS5B amplicons were relatively short, which limited phylogenetic resolution and increased the impact of single-base errors. To mitigate this, sequences were generated bidirectionally, quality-trimmed, and assembled into high-confidence consensus sequences. Despite their length, these regions remain widely used for genotype and subtype discrimination when full-genome data are not available.

In conclusion, our study provided valuable retrospective insights into the status of NPHV in Turkey, where diverse management practices across different ages and breeds of horses existed, allowing for evaluation of risk factors relevant to effective control and prevention strategies. We also employed a combination of approaches to gain foundational understanding of the molecular characteristics of NPHV, which may contribute to future antiviral drug design targeting carrier animals. Moreover, the findings of this study may help shed light on the distribution of NPHV and guide similar investigations in neighbouring countries within the region.

## Data Availability

The obtained partial genome sequences of non-primate hepaciviruses in this study are available under accession numbers PP681161.1–PP681178.1 in GenBank.

## Supplementary Materials

The following supporting information can be downloaded at https://www.mdpi.com/article/10.3390/pathogens14121256/s1, Supplementary Table S1: Detailed information on the viral strains sequenced in this study. Supplementary Table S2: Structural assessment of NS3 and NS5B homology models, evaluating their overall quality.

## Abbreviations

The following abbreviations are used in this manuscript:

5′ UTR	5′ Untranslated Region
BIC	Bayesian Information Criterion
HPV	Hepacivirus
MAVS	Mitochondrial antiviral-signalling protein
MFE	Minimum Free Energy
NPHV	Non-Primate Hepacivirus
RdRP	RNA-dependent RNA Polymerase
SL	Stem-Loop
TRIF	TIR domain-containing adaptor protein inducing interferon beta
