# Supplementary material for: Evaluation of Risk Determinants and Molecular Characterisation for Non-Primate Hepacivirus Infection in Turkish Horses
Source: Pathogens. 2025 Dec 8;14(12):1256. doi: 10.3390/pathogens14121256 (PMC12735631; doi:10.3390/pathogens14121256)
Supplement: Supplementary file 1 [file pathogens-14-01256-s001.zip › pathogens-3993908-supplementary.pdf]

Supplementary Table S1: Detailed information on the viral strains sequenced in this study. The table summarises 15 strains along with the corresponding host horses' age, sex, breed, and sequenced genome regions (5′ UTR, NS3, NS5B) with their accession numbers.

#	Strain Name	Region	Age	Gender	Race	Genome Sequencing
1	Juba8/Sivas	Sivas	4	Male	Indigenous	5′ UTR (PP681170.1), NS3 (PP681161.1)
2	Sibeyi43/Sivas	Sivas	5	Male	Indigenous	5′ UTR (PP681171.1)
3	Balaban47/Balikesir	Balikesir	8	Male	Indigenous	5′ UTR (PP681172.1)
4	Tatar54/Erzurum	Erzurum	10	Male	Indigenous	5′ UTR (PP681173.1)
5	Alkan56/Kayseri	Kayseri	7	Male	Indigenous	5′ UTR (PP681174.1)
6	Karaca62/Ankara	Ankara	7	Male	Indigenous	5′ UTR (PP681175.1)
7	Musa66/Mus	Mus	3	Male	Indigenous	5′ UTR (PP681176.1)
8	Tay84/Hatay	Hatay	8 months	Male	Arabian	5′ UTR (PP681177.1)
9	Kir100/Hatay	Hatay	5	Male	Indigenous	5′ UTR (PP681178.1)
10	Neslihan90/Hatay	Hatay	19	Female	Arabian	NS3 (PP681165.1), NS5B (PP681168.1)
11	Ergulbey51/Balikesir	Balikesir	7	Male	Indigenous	NS3 (PP681164.1)
12	Doru26/Sivas	Sivas	5	Male	Thoroughbred	NS3 (PP681163.1)
13	Efsan22/Sivas	Sivas	10	Male	Indigenous	NS3 (PP681166.1), NS5B (PP681162.1)
14	Alperen60/Ankara	Ankara	14	Male	Indigenous	NS5B (PP681167.1)
15	Araz91/Hatay	Hatay	5	Male	Arabian	NS5B (PP681169.1)

Supplementary Table S2: Supplementary Table S2 presents the structural assessment of NS3 and NS5B homology models, evaluating their overall quality. GMQE values for both proteins indicate reliable models, while QMEANDisCo scores support their accuracy. MolProbity scores, along with low clash scores, suggest minimal steric conflicts. Ramachandran analysis shows that most residues occupy favored regions, with NS5B exhibiting slightly superior stereochemical quality compared to NS3.

#	Protein	GMQE	QMEANDisCo	MolProbity Score	Clash Score	Ramachandran Favoured
1	NS3	0.83	0.79 ± 0.05	1.40	0.74	94.59%
2	NS5B	0.83	0.82 ± 0.05	1.02	1.91	97.68%
